# Roles of cytosolic phospholipase A_2_α in reproductive and systemic toxicities in 2,3,7,8-tetrachlorodibenzo-*p*-dioxin-exposed mice

**DOI:** 10.1007/s00204-017-2081-z

**Published:** 2017-10-17

**Authors:** Nozomi Fujisawa, Wataru Yoshioka, Hiroyuki Yanagisawa, Chiharu Tohyama

**Affiliations:** 1 Laboratory of Environmental Health Sciences, Center for Disease Biology and Integrative Medicine, Graduate School of Medicine, The University of Tokyo, Tokyo, 113-0033 Japan; 2 Department of Public Health and Environmental Medicine, The Jikei University School of Medicine, Tokyo, 105-8461 Japan; 3 Department of Anatomy and Embryology, Faculty of Medicine, University of Tsukuba, Tsukuba, 305-8575 Japan

**Keywords:** cPLA_2_α, Dioxin, Fetal lethality, Hydronephrosis, Fatty liver

## Abstract

Exposure to 2,3,7,8-tetrachlorodibenzo-*p*-dioxin (TCDD) induces a variety of toxicities upon binding of TCDD to aryl hydrocarbon receptor. Although this binding upregulates the synthesis of prostaglandins and their related lipid mediators via cytosolic phospholipase A_2_α (cPLA_2_α), toxicological significance of this signaling pathway remains elusive. Herein, we investigated the roles of cPLA_2_α in TCDD toxicities using cPLA_2_α-null mice. In a first set of experiments, pregnant mice were orally administered TCDD at a dose of 40 μg/kg on gestation day (GD) 12.5, and fetuses were collected on GD 18 for subsequent analyses. The number of live male fetuses of cPLA_2_α-null type was significantly less than that of wild-type in TCDD-exposed litters. TCDD-induced hydronephrosis was more severe in wild-type fetuses than in cPLA_2_α-null fetuses regardless of sex, and kidney expression levels of the inflammatory cytokines interleukin-1β and tumor necrosis factor-α were increased in a cPLA_2_α-dependent manner in TCDD-exposed fetuses. In a second set of experiments, following intraperitoneal administration of TCDD at 50 μg/kg, body weight of the male adult mice was decreased within 2 days in wild-type mice but was not changed in cPLA_2_α-null mice. In addition, TCDD-induced lipid accumulation in the livers of cPLA_2_α-null mice was at an intermediate level compared with TCDD-exposed wild-type and vehicle-control mice. In conclusion, the present results show that cPLA_2_α is involved in TCDD-induced body weight loss, lipid accumulation in the liver, fetal hydronephrosis, and cytokine gene expression, and that the molecular basis of TCDD toxicity differs considerably between target tissues and life stages.

**Electronic supplementary material:**

The online version of this article (doi:10.1007/s00204-017-2081-z) contains supplementary material, which is available to authorized users.

## Introduction

Dioxins are persistent environmental contaminants that are produced unintentionally during combustion and industrial processing, and have become widespread in the environment (Kulkarni et al. 2008). Among many dioxin congeners, 2,3,7,8-tetrachlorodibenzo-*p*-dioxin (TCDD), which binds strongly to the aryl hydrocarbon receptor (AHR), has been used as a prototypical congener in experimental studies (Van den Berg et al. 2006). Laboratory animal studies of TCDD demonstrate various modes of toxicity, including teratogenicity, reproductive toxicity, immune dysfunction, carcinogenicity, and neurobehavioral disorders (Pohjanvirta and Tuomisto 1994). AHR is classified as a protein of the basic helix-loop-helix/period-ARNT-single-minded (bHLH/PAS) family and functions as a cytosolic transcription factor (Burbach et al. 1992). Upon activation of AHR by ligands, the AHR–ligand complex translocates from the cytoplasm to the nucleus with assistance of cofactors, and subsequently forms a heterodimer with the transcription factor AHR nuclear translocator (ARNT). Heterodimerized AHR complexes then bind to the AHR responsive element (also named xenobiotic responsive element or dioxin responsive element), leading to transcriptional activation of AHR target genes, such as those that encode CYP1A1 (Mimura and Fujii-Kuriyama 2003). Because AHR-null mice that are produced by deleting one of the three exons are resistant to dioxin insult (Fernandez-Salguero et al. 1996; Mimura et al. 1997; Peters et al. 1999), AHR is considered pivotal to TCDD toxicities.

Levels of prostaglandins and their related lipid mediators are increased in an AHR-dependent manner following TCDD exposure in kidneys (Nishimura et al. 2008) and in several other organs (Bui et al. 2012). Recently, in vivo experiments revealed that prostaglandin E_2_ plays a pivotal role in TCDD-induced neonatal hydronephrosis in mouse pups. Specifically, TCDD exposure upregulated the expression of the key prostaglandin E_2_ synthesis enzymes, cytosolic phospholipase A_2_α (cPLA_2_α), cyclooxygenase-2 (COX-2), and microsomal prostaglandin E synthase 1 (mPGES-1), leading to excessive production of prostaglandin E_2_ (PGE_2_) in kidneys from mouse neonates (Nishimura et al. 2008; Yoshioka et al. 2012, 2014). COX-2 (Nishimura et al. 2008) and mPGES-1 (Yoshioka et al. 2012) were shown to play essential roles in the development of TCDD-induced hydronephrosis in neonatal mice, and cPLA_2_α was found to play a predominant role in TCDD-induced hydronephrosis by upregulating COX-2 and mPGES-1 (Yoshioka et al. 2014).

cPLA_2_α catalyzes hydrolysis of glycerophospholipids in cell membranes, leading to release of lysophospholipids and fatty acids such as arachidonic acid (AA) (Murakami et al. 2011). In addition, cPLA_2_α was shown to be transcriptionally (Kinehara et al. 2009) or enzymatically activated (Dong and Matsumura 2008) following TCDD exposure, and the produced AA was subsequently converted into prostaglandins, thromboxane, and leukotrienes. Accordingly, cPLA_2_α influences various biological phenomena, including reproduction, immune responses, and development of cancer and atherosclerosis, through the production of prostaglandins, thromboxane, and leukotrienes (Leslie 2015). Thus, diverse responses to TCDD-mediated cPLA_2_α activation may manifest in a variety of TCDD toxicities. Herein, we characterized the roles of cPLA_2_α in various TCDD toxicities in fetal and adult mice.

## Materials and methods

### Reagents and chemicals

TCDD (purity, > 99.1%) was purchased from AccuStandard (New Haven, CT, USA) and was dissolved in corn oil containing 2% *n*-nonane. All other reagents were purchased from Wako Pure Chemicals (Osaka, Japan), unless otherwise stated.

### Animals and treatments

All animal experimental protocols were approved by the Animal Care and Use Committee of the University of Tokyo in accordance with Institutional Guidelines for Animal Experimentation, the Japanese Government Law concerning the Protection and Control of Animals, and Japanese Government Notification of Feeding and Safekeeping of Animals. Mice deficient in *Pla2g4a*, which encodes cPLA_2_α, were kindly provided by Dr. Takao Shimizu (The University of Tokyo) (Uozumi et al. 1997), and hetero- and homozygous mice were designated cPLA_2_α^+/−^ and cPLA_2_α^−/−^, respectively. Wild-type littermates were designated cPLA_2_α^+/+^. These cPLA_2_α deficient mice were backcrossed with C57BL/6 J mice more than 12 times in the Shimizu laboratory, and then more than six times in the Tohyama laboratory. Genotypes of the mice were determined using PCR with genomic DNA as previously described (Uozumi et al. 1997).

**Fig. 1:**
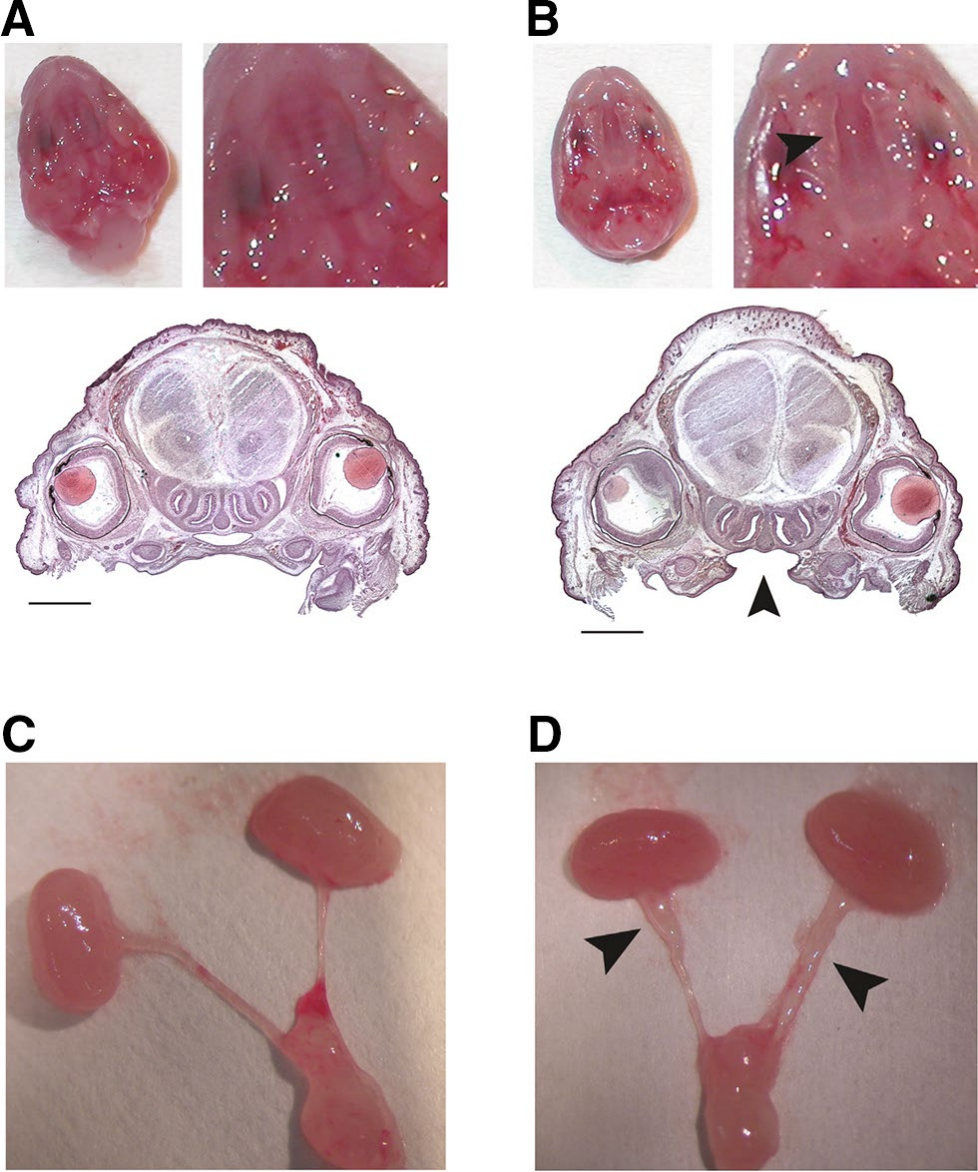
TCDD-induced cleft palate and hydroureter in fetuses. Pregnant cPLA_2_α^+/−^ mice were administered TCDD at 0 or 40 μg/kg body weight on GD 12.5 and fetuses were analyzed on GD 18.5. Representative photographs of normal palate (**a**), cleft palate (**b**), normal ureter (**c**), and hydroureter (**d**); arrowheads in **b** and **d** indicate failure of palatal shelves to fuse and marked dilation of ureters, respectively; Bar = 1.0 mm

To assess TCDD toxicities in fetuses, male and female cPLA_2_α^+/−^ mice were mated overnight, and females were checked for vaginal plugs the following morning. The day on which the presence of a vaginal plug was confirmed was designated gestational day (GD) 0. Pregnant female mice were then administered TCDD via gavage at doses of 0 or 40 μg/kg body weight on GD 12.5. These experimental conditions including the dose, timing, and route of administration were adopted from a previous study on TCDD-induced fetal toxicity (Mimura et al. 1997). Female mice were then killed on GD 18.5 by cervical dislocation followed by Caesarean section and fetal mortality was assessed according to the number of live fetuses per litter. Live fetuses were immersed in ice-cold phosphate-buffered saline (PBS) and kidneys (left) were then dissected, snap-frozen in liquid nitrogen, and stored at −80 °C for RNA extraction. Fetuses were examined stereoscopically for the presence of hydroureter, dilated ureter filled with urine, and cleft palate (Fig. 1) as previously described (Bryant et al. 2001). Kidneys (right) and heads (including palate) were fixed in 10% neutral buffered formalin for histological analyses.

In separate experiments, TCDD toxicities were assessed in adult mice (10–12-week-old) from cPLA_2_α^+/+^ (26.0 ± 1.0 g in body weight) and cPLA_2_α^−/−^ (24.8 ± 1.1 g) groups following a single intraperitoneal injection of TCDD (0 or 50 μg/kg body weight). The TCDD dose was selected according to previous reports in which liver injury (Yoshioka et al. 2011) and other short-term toxicity (Pohjanvirta and Tuomisto 1994) were observed. The day of injection was designated Day 0, and blood was collected from the caudal vein every other day to obtain plasma. Mice were killed on Day 8 and liver and thymus tissues were collected from each mouse and weighed. Organs were then snap-frozen in liquid nitrogen and stored at −80 °C for extraction of RNA and protein. Portions of liver lobes were fixed in 10% neutral buffered formalin for histological analyses.

### Histopathology

Fixed tissues (kidney and palate of fetuses, and liver of adults) were cryoprotected in 20% sucrose solution overnight and were embedded in O.T.C. compound (Sakura Finetek Japan, Tokyo, Japan). Embedded tissues were then frozen on an aluminum block that had been submerged in liquid nitrogen. Sliced sections (5-μm thickness) of kidneys and livers were then stained with hematoxylin and eosin (Muto Pure Chemicals, Tokyo, Japan) and Oil Red O (Muto Pure Chemicals), respectively.

**Fig. 2:**
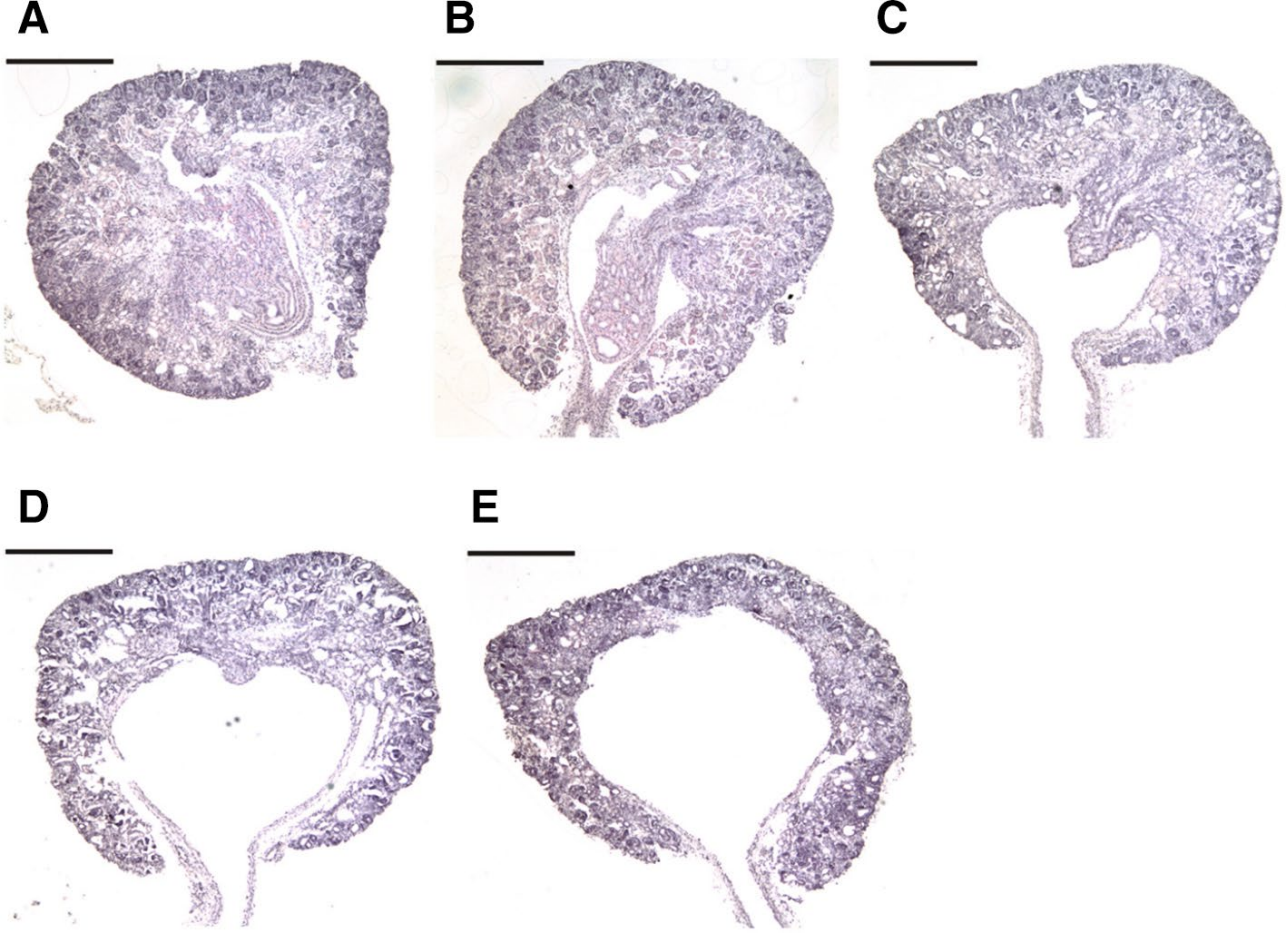
TCDD-induced hydronephrosis in fetuses. Pregnant cPLA_2_α^+/−^ mice were administered TCDD at 0 or 40 μg/kg body weight on GD 12.5 and fetuses were analyzed on GD 18.5. Representative diagnostic photographs are shown as follows: **a** severity score 0, normal kidney with highly developed papilla that fill the pelvic space; **b** severity score 1, kidneys with slight pelvic space; **c** severity score 2, kidneys with reduced papillary sizes and considerable pelvic spaces; **d** severity score 3, kidneys with limited papilla, dilated pelvic spaces, and thinned renal parenchyma; **e** severity score 4, kidneys with deteriorated papilla and substantially thinned renal parenchyma; Bar = 1.0 mm

The severity of hydronephrosis was scored according to previously described criteria (Bryant et al. 2001). Scores of 0 and 4 were assigned to kidneys with no signs of hydronephrosis and the most severe degrees of hydronephrosis, respectively (Fig. 2). Kidneys with scores of ≥ 2 were considered diagnostic of hydronephrosis (Bryant et al. 2001; Theobald and Peterson 1997).

### RNA extraction and quantitative reverse transcription polymerase chain reaction (RT-PCR)

Tissue specimens (kidneys from fetuses and livers from adults) were homogenized in TRIzol reagent (Thermo Fisher Scientific, MA, USA) using a Polytron homogenizer (Kinematica, Luzern, Switzerland) following the manufactures’ instructions. Aqueous phases of homogenates were further purified using an RNeasy Mini Kit (Qiagen, Hilden, Germany) for mRNA quantification, and using a miRNeasy mini Kit (Qiagen) for miRNA quantification. RNAs were then reverse-transcribed using a PrimeScript RT reagent Kit (Takara Bio, Kusatsu, Japan) with oligo-dT and dN_6_ primers for mRNAs, and a Mir-X miRNA First-Strand Synthesis Kit (Takara Bio) for miRNAs. Quantitative gene expression analyses were performed using the Thunderbird SYBR qPCR Mix (Toyobo, Osaka, Japan), a LightCycler System (Roche Molecular Biochemicals, IN, USA), and the primers listed in Supplementary Table 1. Amplification specificities were determined using melting curve analyses for each PCR. Gene expression levels were calculated using the ΔCt method (Schmittgen and Livak 2008) and mRNAs and miRNAs were normalized to cyclophilin B and U6 snRNA, respectively.

### Protein extraction and immunoblotting

Intermediate and phenol phases of TRIzol RNA extraction homogenates were further processed to extract proteins following the manufacturer’s instructions with a slight modification. Briefly, proteins in the intermediate and phenol phases were purified by ethanol precipitation to remove DNA, were precipitated with isopropanol, and were then resuspended in a solution containing 7 M urea, 2 M thiourea, 3% CHAPS, and 1% Triton X-100. Subsequently, equal volumes of 2 × SDS solution containing 0.1 M Tris–Cl (pH 6.8), 4% SDS, 20% glycerol, and 12% 1-thioglycerol were added to protein extracts. Proteins were then separated on 8% SDS–polyacrylamide gels and were transferred to polyvinylidene difluoride membranes (Cat. No. ISEQ07850, Merck, Darmstadt, Germany). Immunoblots were performed using a primary rabbit polyclonal antibody against adipophilin (Cat. No. NB110-40878; Novus Biologicals, CO, USA), against CYP1A1 (Cat. No. CSB-PA001929; Flarebio, Baltimore, USA), and a primary HRP-conjugated rabbit monoclonal antibody against β-Actin (Cat. No. 5125; Cell Signaling Technology Japan, K.K., Tokyo, Japan). The anti-adipophilin antibody on membranes was detected using HRP-conjugated IgG detector (Cat. No. T7122A; Takara Bio) and protein bands were visualized using chemiluminescence (Cat. No. WBLUF0500; Merck). Adipophilin protein abundance was quantified by band intensity analyses and was normalized to that for β-actin using a LAS3000 mini system (Fujifilm, Tokyo, Japan).

### Alanine aminotransferase activity assay

Alanine aminotransferase (ALT) activities in plasma samples were determined using a blood biochemistry analyzer (Dri-Chem, model 7000 V, Fujifilm).

### Statistical analysis

To minimize litter effects, fetal data were classified according to sex, genotype, and TCDD dose, and were averaged on a within-litter basis and then among litters. The number of fetuses per litter was compared using Wilcoxon's tests with Bonferroni's correction. Severities of hydronephrosis were compared using *χ* ^2^ tests. Tissue weights and mRNA expression levels were compared using ANOVA followed by Tukey's multiple comparisons. Differences in body weights and ALT were identified using repeated measures ANOVA followed by *t* tests with Bonferroni's correction. Data are shown as means ± standard errors of the mean (SEM) and differences were considered significant when *p* < 0.05.

## Results

### Relationships between cPLA_2_α genotypes and the number of live fetuses following TCDD exposures

No dams died by GD 18.5 in either control or TCDD treatment groups. On GD 18.5, the number of live fetuses of either sex in the vehicle control group was comparable between cPLA_2_α^−/−^ and cPLA_2_α^+/+^ genotypes (Table 1), and the ratio between cPLA_2_α^+/+^, cPLA_2_α^+/−^, and cPLA_2_α^−/−^ genotypes did not deviate from Mendel’s law of segregation. These results indicate that cPLA_2_α loss was not independently involved in fetal death by GD 18.5. In contrast, TCDD exposure caused considerable deviations in genotype ratios among male fetuses from Mendel’s law of segregation. In TCDD-exposed litters, the number of live male fetuses of the cPLA_2_α^−/−^ genotype was significantly less than that of the cPLA_2_α^+/+^ genotype (0.53 ± 0.16 vs 1.37 ± 0.21; Wilcoxon's signed rank test, *p* = 0.011). TCDD exposure tended to reduce the number of live male fetuses of cPLA2α^−/−^ genotype compared with that of vehicle-control male cPLA2α^−/−^ fetuses (0.53 ± 0.16 vs. 1.13 ± 0.29; Wilcoxon's rank sum test, *p* = 0.059). No significant differences in the number of live female fetuses was found following TCDD exposure regardless of cPLA_2_α genotype (Table 1). These results suggest that the absence of cPLA_2_α results in TCDD-induced reduction in the number of live fetuses among male animals only.

**Table 1:** Number of male and female fetuses on GD 18.5

TCDD dose (μg/kg)	Number of dams	cPLA_2_α genotype	Male fetuses		Female fetuses	
			Number	Litter size	Number	Litter size
40	30	+/+	41	1.37 ± 0.21	28	0.93 ± 0.14
		+/−	42	1.40 ± 0.22	57	1.90 ± 0.24
		−/−	16	0.53 ± 0.16^a^	28	0.93 ± 0.20
0	16	+/+	16	1.00 ± 0.22	15	0.94 ± 0.23
		+/−	23	1.44 ± 0.24	26	1.63 ± 0.24
		−/−	18	1.13 ± 0.29	10	0.63 ± 0.22

Pregnant cPLA_2_α^+/−^ mice were administered TCDD (40 μg/kg) or corn oil on GD 12.5. The number of fetuses was compared between sexes, genotypes, and TCDD-doses, and were averaged on a litter basis and then among litters. Values are shown as means ± standard errors of the mean

^a^Significantly different from TCDD-exposed male cPLA_2_α^+/+^ fetuses; Wilcoxon's signed rank test, *p* < 0.05

### Roles of cPLA_2_α in TCDD-induced teratogenicity

Cleft palate and hydronephrosis are hallmarks of TCDD-induced teratogenesis (Mimura et al. 1997; Moriguchi et al. 2003; Theobald et al. 2003), and hydroureter was reportedly associated with TCDD-induced fetal hydronephrosis (Abbott et al. 1987). To investigate whether these toxicity phenotypes depend on cPLA_2_α, we compared cPLA_2_α^−/−^ and cPLA_2_α^+*/*+^ fetuses. In these experiments, the incidence of cleft palate exceeded 85% among TCDD-exposed male and female fetuses of both cPLA_2_α genotypes (Table 2), indicating that cPLA_2_α does not play a major role in TCDD-induced cleft palate.

**Table 2:** Incidence and severity of hydronephrosis and cleft palate in fetuses on GD 18.5

TCDD dose (µg/kg)	cPLA_2_α genotype	*n* [fetus (dam)]^a^	Cleft palate incidence (%)	Hydronephrosis						
				Severity						Incidence (%)
				0	1	2	3	4	Average	
Male										
40	+/+	41 (25)	94.0 ± 5.2	4	5	15	12	5	2.21 ± 0.20	75.0 ± 8.2
	−/−	16 (11)	100 ± 0	2	3	5	6	0	1.83 ± 0.27	65.2 ± 13.6
0	+/+	16 (11)	0	11	5	0	0	0	0.33 ± 0.14	0
	−/−	18 (10)	0	13	4	1	0	0	0.42 ± 0.20	10.0 ± 10.0
Female										
40	+/+	28 (22)	89.4 ± 8.0	3	1	9	7	8	2.55 ± 0.29	81.8 ± 8.4
	−/−	29 (16)	90.6 ± 9.1	7	3	8	10	1	1.79 ± 0.30	63.5 ± 10.7
0	+/+	14 (9)	0	11	3	0	0	0	0.22 ± 0.12	0
	−/−	10 (7)	0	7	3	0	0	0	0.26 ± 0.14	0

Diagnoses of hydronephrosis and cleft palate were made on a litter basis. Kidneys were diagnosed with hydronephrosis when scores were grade 2 and over. Values are shown as means ± standard errors of the mean

^a^Dams that did not have fetuses were not included

Administration of TCDD to pregnant mice led to a high incidence of hydronephrosis in male and female fetuses (Table 2), as has been shown in previous studies (Couture-Haws et al. 1991; Couture et al. 1990; Moore et al. 1973). In addition, the severity of hydronephrosis was associated with cPLA_2_α genotype, with more prevalent severity scores of 4 in cPLA_2_α^+*/*+^ fetuses (males, 5 in 41; females, 8 in 28) than in cPLA_2_α^−/−^ fetuses (males, 0 in 16; females, 1 in 29). Average severity scores for TCDD-exposed groups also tended to be higher in cPLA_2_α^+*/*+^ fetuses (male, 2.21 ± 0.20; female, 2.55 ± 0.29) than in the corresponding cPLA_2_α^−/−^ fetuses (male, 1.83 ± 0.27; female, 1.79 ± 0.30). Spontaneous mild hydronephrosis (score 2) was observed in one male fetus in the vehicle-control group (Table 2). Hydroureter was observed in more than 90% of pups exposed to TCDD (Fig. 1c, d) and did not depend on cPLA_2_α genotype (Table 3).

**Table 3:** Incidence of hydroureter in fetuses on GD18.5

TCDD dose (µg/kg)	cPLA_2_α genotype	*n* [fetus (dam)]^a^	Hydroureter incidence^b^ (%)
Male			
40	+/+	27 (14)	100
	−/−	12 (7)	92.9 ± 7.1
0	+/+	13 (9)	0
	−/−	17 (9)	0
Female			
40	+/+	15 (13)	92.3 ± 7.7
	−/−	18 (11)	90.0 ± 10.0
0	+/+	14 (9)	0
	−/−	10 (7)	0

^a^Dams that did not have fetuses were not included

^b^Calculated on a litter basis

### Gene expression in kidneys of TCDD-exposed mouse fetuses

**Fig. 3:**
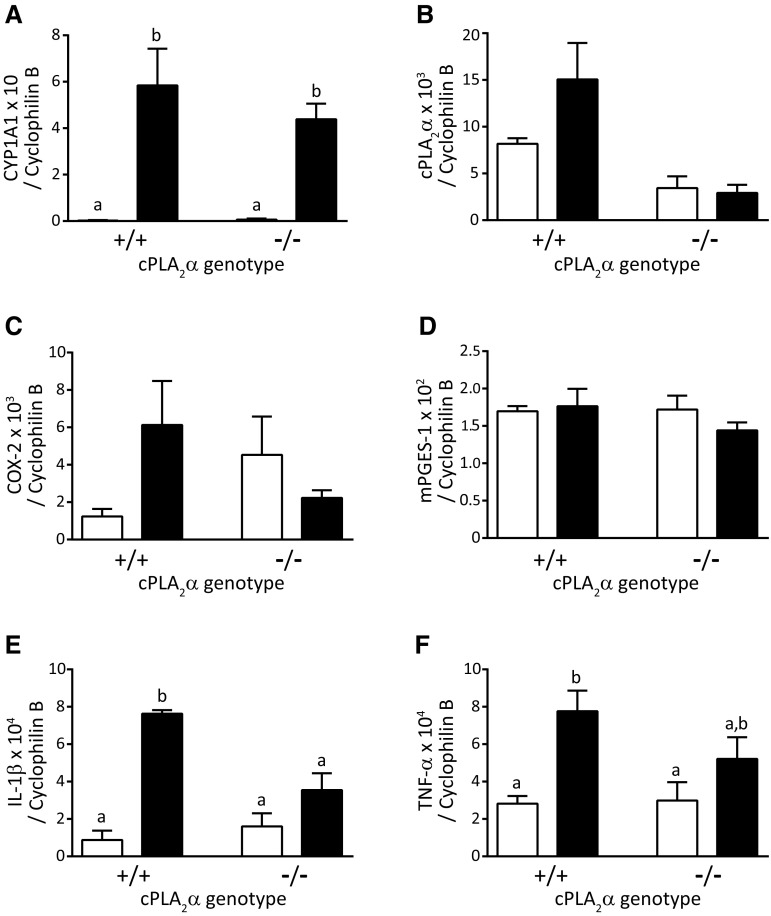
Gene expression levels in kidneys from TCDD-exposed cPLA_2_α^+/+^ and cPLA_2_α^−/−^ mice fetuses. CYP1A1 (**a**), cPLA_2_α (**b**), COX-2 (**c**), mPGES-1 (**d**), IL-1β (**e**), and TNF-α (**f**) mRNA levels in kidneys from TCDD-exposed cPLA_2_α^+/+^ and cPLA_2_α^−/−^ male fetuses on GD 18.5; fetuses were collected from pregnant cPLA_2_α^+/−^ dams that were administered TCDD at 0 or 40 μg/kg body weight on GD 12.5. Values were normalized to cyclophilin B expression. Values and bars indicate means ± standard errors of the mean (SEM; *n* = 3). Histograms with different letters indicate significant differences by Tukey’s post hoc test. No statistical comparison was performed for cPLA_2_α^−/−^ pups expressing truncated cPLA_2_α

To investigate the molecular basis of TCDD-induced fetal hydronephrosis, we analyzed expression of genes in kidneys on GD 18.5. CYP1A1 mRNA abundance is well-known to reflect AHR transactivation capacity (Mimura and Fujii-Kuriyama 2003) and was significantly increased in the TCDD-exposed group compared with the vehicle-control group regardless of cPLA_2_α genotype (Fig. 3a).

In mRNA analyses of genes encoding enzymes for prostaglandin synthesis, cPLA_2_α mRNA abundance in the TCDD-exposed cPLA_2_α^+/+^ group tended to be higher than in the vehicle-control cPLA2α^+/+^ group, but this difference was not statistically significant (Fig. 3b). The minimal expression of cPLA_2_α mRNA in cPLA_2_α^−/−^ fetuses reflected the presence of truncated cPLA_2_α that lacks enzyme activity (Uozumi et al. 1997). Among cPLA_2_α^+*/*+^ fetuses, COX-2 mRNA abundance in the TCDD-exposed group tended to be higher than that in the vehicle-control group (Fig. 3c). Although the lowest value for COX-2 mRNA abundance in the TCDD-exposed group exceeded the highest value in the vehicle-control group, the difference was not statistically significant owing to the large variation of the values in the TCDD-exposed group. Among cPLA_2_α^−/−^ fetuses, COX-2 mRNA abundance in the TCDD-exposed group tended to be reduced compared with that in the vehicle-control group (Fig. 3c). Abundance of mPGES-1 mRNA was not affected in TCDD-exposed fetuses, regardless of cPLA_2_α genotype (Fig. 3d).

Abundance of mRNA of interleukin (IL)-1β, an inflammatory cytokine, was significantly greater in the TCDD-exposed cPLA_2_α^+/+^ group than in the vehicle-control cPLA_2_α^+/+^ group (Fig. 3e). On the other hand, there was no significant difference between the TCDD-exposed and vehicle-control cPLA_2_α^−/−^ groups (Fig. 3e). In addition, IL-1β mRNA abundance in the TCDD-exposed cPLA_2_α^−/−^ group was significantly less than in the TCDD-exposed cPLA_2_α^+/+^ group. These results demonstrate that TCDD-induced IL-1β expression in fetal kidney depends on cPLA_2_α. In accordance, tumor necrosis factor (TNF)-α mRNA had essentially the same expression pattern as IL-1β mRNA, albeit with less clear difference between the TCDD-exposed cPLA_2_α^−/−^ and cPLA_2_α^+/+^ groups (Fig. 3f).

### Effects of TCDD on body and tissue weights in adult mice

**Fig. 4:**
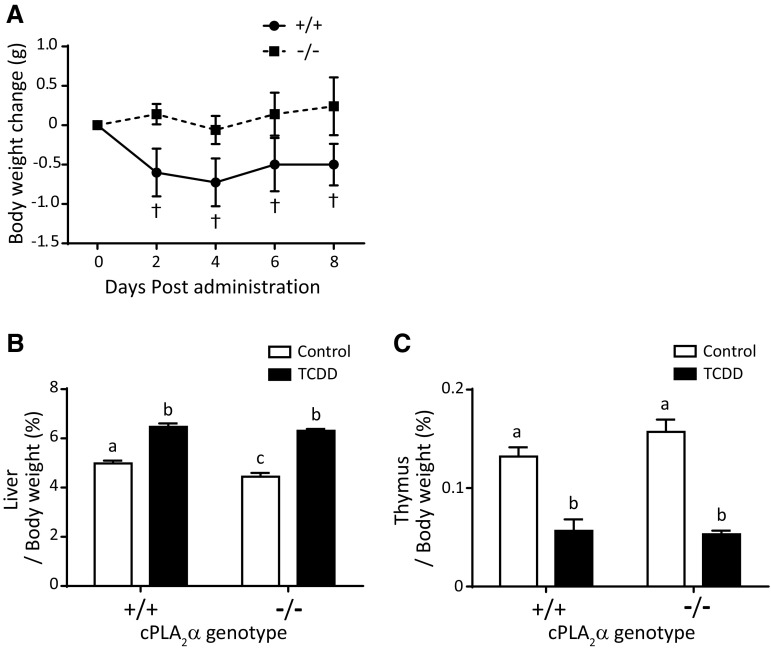
TCDD toxicity phenotypes in adult mice. Body weights (**a**) were measured every other day (Days 0–8) following administration of TCDD (50 μg/kg body weight) or corn oil on Day 0. Mice were killed on Day 8. Liver (**b**) and thymus (**c**) weights were those normalized to final body weights. Values and bars indicate mean ± SEM (*n* = 3 − 5); *p < 0.05 and **p < 0.01, significant differences within the same genotype

During the 8 days following TCDD administration at a dose of 50 μg/kg, body weight of adult male mice in the cPLA_2_α^+/+^ group was significantly reduced, but remained unchanged in the cPLA_2_α^−/−^ group (Fig. 4a). At Day 8 post-administration, relative liver to body weights of TCDD-exposed cPLA_2_α^+/+^ and cPLA_2_α^−/−^ groups were significantly larger than those of the vehicle-treated groups, in which relative liver weight of cPLA_2_α^−/−^ group was slightly but significantly smaller than that of cPLA_2_α^+/+^ group (Fig. 4b). Relative thymus to body weights decreased at Day 8 post-TCDD administration in a cPLA_2_α genotype-independent manner (Fig. 4c).

### TCDD-induced damage to livers of adult male mice

**Fig. 5:**
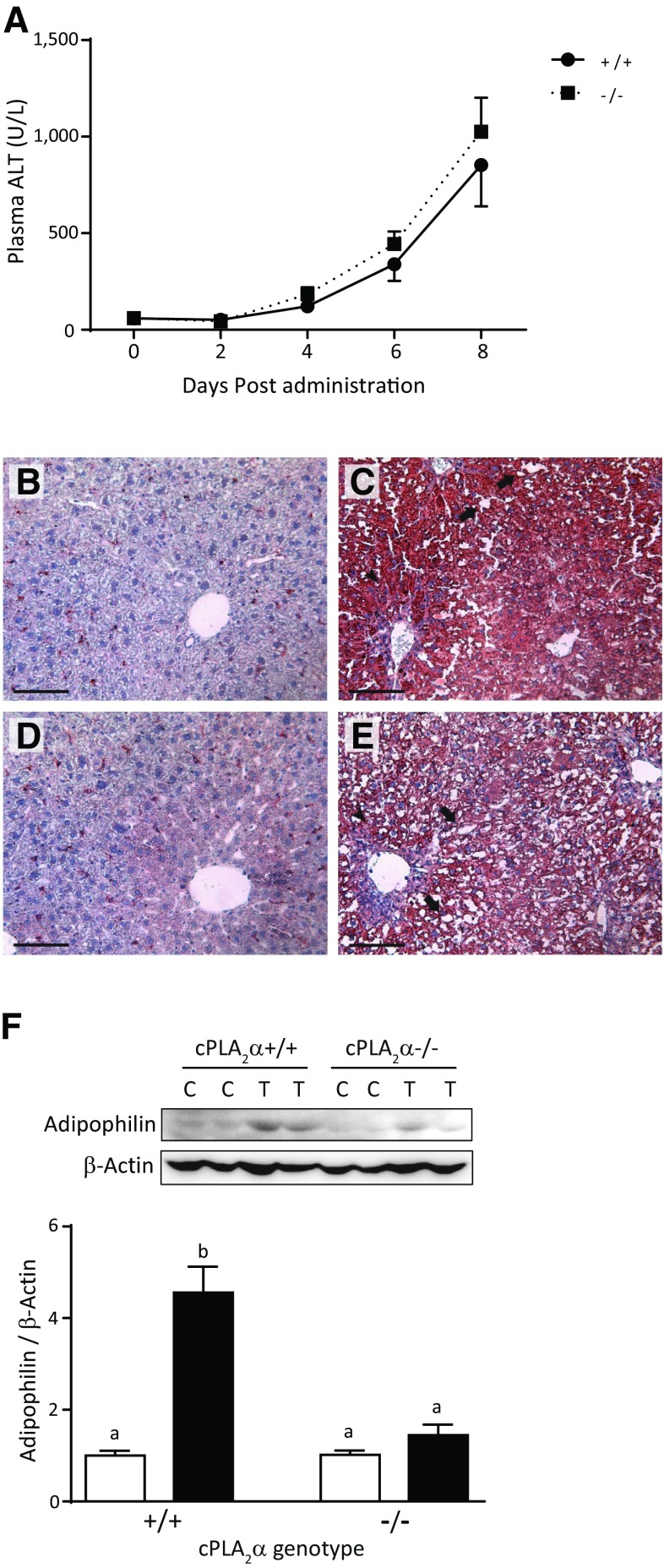
TCDD-induced hepatic damage in adult mice. **a** Time-course of plasma ALT levels in male cPLA_2_α^+/+^ and cPLA_2_α^−/−^ mice following administration of TCDD at 50 μg/kg body weight. Values and bars indicate mean ± SEM (*n* = 8–9). **b**–**e** Representative photographs of Oil Red O staining of liver sections from cPLA_2_α^+/+^ and cPLA_2_α^−/−^ mice on Day 8 post-TCDD administration; adult cPLA_2_α^+/+^ (**b**, **c**) and cPLA_2_α^−/−^ (**d**, **e**) mice were injected intraperitoneally with TCDD at doses of 0 (**b**, **d**) or 50 (**c**, **e**) μg/kg body weight. Arrows, vacuolization; arrowheads, inflammatory cell infiltration; Bar = 100 μm; **f** a representative gel image of hepatic adipophilin protein in western blot analyses (upper panel). The graph (lower panel) indicates relative adipophilin protein expression normalized to that of β-actin. Values and bars indicate mean ± SEM (*n* = 4). Different letters on histograms indicate significant differences by Tukey’s post hoc test

TCDD treatments significantly increased plasma ALT levels in adult male mice at 6–8 days post-administration, being indifferent to the cPLA_2_α genotype (Fig. 5a). Histological examinations with Oil Red O neutral lipid staining revealed that TCDD-exposed mice developed fatty liver, as characterized by increased neutral lipid contents, vacuolization, and infiltration of inflammatory cells at Day 8 (Fig. 5c, e). In contrast, vehicle-control mice did not develop histopathological liver abnormalities (Fig. 5b, d). Hematoxylin and eosin staining analyses confirmed these pathological changes in TCDD-exposed livers (Supplementary Fig. 1). Because neutral lipid staining in the livers in the TCDD-exposed cPLA_2_α^−/−^ group (Fig. 5e) appeared to be weaker than that in the cPLA_2_α^+/+^ group (Fig. 5c), we examined the expression of adipophilin, which is a protein component of lipid droplets (Motomura et al. 2006). Adipophilin protein abundance in the TCDD-exposed cPLA_2_α^+/+^ group was 4.56 times greater than that in the vehicle-control mice (*p* < 0.01), but was increased by only 1.42 times in the TCDD-exposed cPLA_2_α^−/−^ group compared with the corresponding control group (*p* = 0.89; Fig. 5f). Furthermore, adipophilin protein abundance in the TCDD-exposed cPLA_2_α^−/−^ group was significantly less than in the TCDD-exposed cPLA_2_α^+/+^ group (Fig. 5f), indicating that TCDD-induced adipophilin expression depends on cPLA_2_α.

### TCDD-induced gene expression in the livers of cPLA_2_α^+/+^ and cPLA_2_α^−/−^ mice

**Fig. 6:**
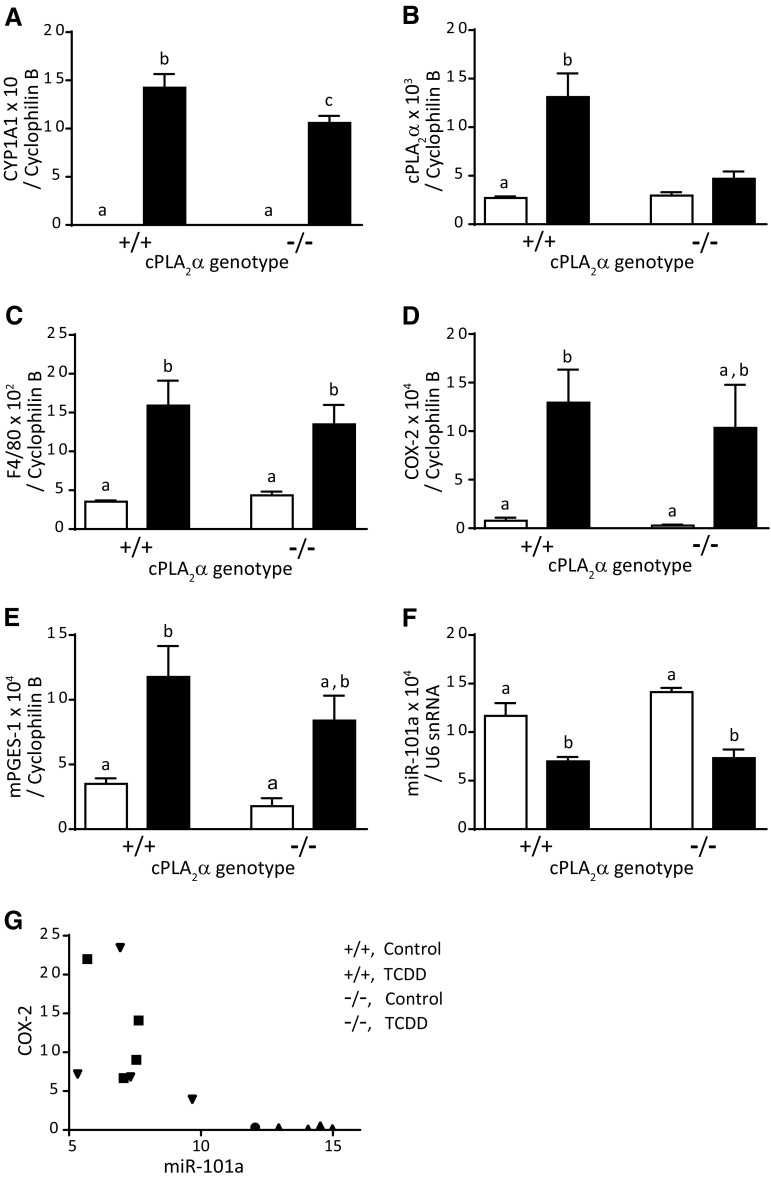
CYP1A1 (**a**), cPLA_2_α (**b**), F4/80 (**c**), COX-2 (**d**), and mPGES-1 (**e**) mRNA, and miR-101a (**f**) expression in livers of adult male cPLA_2_α^+/+^ and cPLA_2_α^−/−^ mice at 8 days post-administration of TCDD (50 μg/kg body weight) or vehicle. **g** Association of COX-2 mRNA and miR-101a expression levels; values for mRNAs and miR-101a are normalized to cyclophilin B mRNAs and U6 snRNA expression levels, respectively. Values and bars indicate mean ± SEM (*n* = 4). Histograms with different letters indicate significant differences by Tukey’s post hoc test. No comparisons were performed for cPLA_2_α^−/−^ pups expressing truncated cPLA_2_α

CYP1A1 mRNA abundance in the vehicle-control cPLA_2_α^−/−^ group was not different from that of the vehicle-control cPLA_2_α^+/+^ group (Fig. 6a). CYP1A1 mRNA abundance in the TCDD-exposed cPLA_2_α^+/+^ group was significantly greater than that in two vehicle-control groups, or vehicle-control cPLA_2_α^+/+^ and cPLA_2_α^−/−^ groups. Similarly, the abundance in the TCDD-exposed cPLA_2_α^−/−^ group was significantly greater than that in the vehicle-control groups. In addition, the abundance in the TCDD-exposed cPLA_2_α^−/−^ group was significantly less than that in the TCDD-exposed cPLA_2_α^+/+^ group. These results indicate that the basal expression of CYP1A1 is independent of cPLA_2_α and that TCDD-induced upregulation of CYP1A1 partially depends on cPLA_2_α. The extent of this dependency was estimated to be 25% according to the comparison of an increase in CYP1A1 mRNA abundance of the TCDD-exposed cPLA_2_α^−/−^ group from the basal (an increase in the abundance = 10.6) with that of the TCDD-exposed cPLA_2_α^+/+^ group (an increase in the abundance = 14.2). Abundance of CYP1A1 protein in the TCDD-exposed cPLA_2_α^+/+^ and cPLA_2_α^−/−^ groups were not significantly different (Supplementary Fig. 2d). Abundance of mRNAs of other AHR target genes, such as AHRR, CYP1B1, and Nqo1, showed essentially the same expression pattern as CYP1A1 mRNA, with significant differences in CYP1B1 and Nqo1 mRNAs, but not in AHRR mRNA (Supplementary Fig. 2). TCDD administration also substantially increased cPLA_2_α mRNA abundance in the cPLA_2_α^+/+^ groups (Fig. 6b). Moreover, mRNA abundance of the macrophage marker F4/80 was increased following TCDD exposure in a cPLA_2_α independent manner (Fig. 6c). COX-2 mRNA was significantly more abundant in the TCDD-exposed cPLA_2_α^+/+^ group than in the vehicle-control groups (Fig. 6d). In contrast, COX-2 mRNA abundance in the TCDD-exposed cPLA_2_α^−/−^ group was not significantly different from that in the vehicle-control groups or that in the TCDD-exposed cPLA_2_α^+/+^ group, and mean value of the abundance was at an intermediate level between those in TCDD-exposed cPLA_2_α^+/+^ and vehicle control groups (Fig. 6d). mPGES-1 mRNA had essentially the same expression pattern as COX-2 mRNA (Fig. 6e). Abundance of miR-101a, which regulates COX-2 expression (Chakrabarty et al. 2007; Strillacci et al. 2009; Tanaka et al. 2009), was significantly decreased following TCDD exposure, regardless of cPLA_2_α genotype (Fig. 6f). Consistent with roles as a negative regulator of COX-2, miR-101a abundance was inversely correlated with COX-2 mRNA abundance (Fig. 6g) with a correlation coefficient of −0.71.

## Discussion

In this study, we investigated the roles of cPLA_2_α in the toxicities of TCDD using cPLA_2_α^+/+^ and cPLA_2_α^−/−^ mice, and showed that genetic ablation of cPLA_2_α neither ameliorates or exacerbates TCDD-induced cleft palate, hydroureter, hepatomegaly, or thymic atrophy. These results suggest that cPLA_2_α does not play significant roles in these TCDD toxicities. However, cPLA_2_α was involved in TCDD-induced body weight loss, lipid accumulation in the liver, fetal hydronephrosis, gene expression in the fetal kidney and in the adult liver, and decrease in the number of live fetuses. The potential roles of cPLA_2_α in these TCDD-induced phenomena are discussed below.

TCDD exposure reduced body weight of adult cPLA_2_α^+/+^ mice (Fig. 4a). In our previous study (Yoshioka et al. 2011), body weight of C57BL/6J mice was significantly reduced 2 days post-TCDD (50 μg/kg) administration and thereafter. A similar observation was reported by a previous study (Matsumura et al. 1997), showing reduction in body weight within a few days following TCDD administration (115 μg/kg). These independent observations demonstrate that TCDD induces a decrease in body weight within a few days post-administration. However, because the body weight reduction was slight, the reduction by itself is not thought to have adverse impacts on TCDD-exposed mice, but could be an early sign of wasting syndrome, which ultimately leads to death following continuous decreases in body weight (Linden et al. 2010). The present experiments showed that cPLA_2_α plays an indispensable role in TCDD-induced body weight loss (Fig. 4a), and is upregulated in the liver by TCDD treatments (Fig. 6b). In a previous study, SRC tyrosine kinase also reportedly mediated TCDD-induced body weight loss (Matsumura et al. 1997) following activation by arachidonic acid (AA), which is produced by cPLA_2_α (Dong and Matsumura 2008). Taken together, these data suggest that the cPLA_2_α/AA/SRC pathway is involved in the onset of TCDD-induced body weight loss. On the other hand, CYP1A1 was also reported to have a role in the TCDD-induced body weight reduction (Uno et al. 2004). In the present study, CYP1A1 mRNA induction in cPLA_2_α^−/−^ mice was slightly lower than that in cPLA_2_α^+/+^ mice (Fig. 6a), which might explain the absence of TCDD-induced body weight loss in cPLA_2_α^−/−^ mice. In addition, TCDD-inducible poly(ADP-ribose) polymerase (Tiparp), another gene inducible by TCDD, has a role in protecting from TCDD-induced body weight loss (Ahmed et al. 2015). Further studies will clarify the relation of cPLA_2_α/AA/SRC, CYP1A1, and Tiparp pathways in TCDD-induced weight loss or wasting syndrome.

TCDD-induced liver damage is a complex phenomenon involving hepatomegaly, inflammation, and lipid accumulation. Moreover, genetic ablation of cPLA_2_α significantly suppressed TCDD-induced increase in adipophilin (Fig. 5f), which is a lipid droplet protein (Motomura et al. 2006). Hence, cPLA_2_α likely contributes to lipid accumulation, as indicated by suppressed neutral lipid staining in the absence of cPLA_2_α (Fig. 5c, e). The major contribution of cPLA_2_α to lipid accumulation has also been reported in a high fat diet-induced mouse model of fatty liver (Ii et al. 2009), suggesting that cPLA_2_α has important roles in the development of fatty liver due to various causes. In contrast, cPLA_2_α played only minimal roles in inflammatory reactions, hepatomegaly, and miR-101a-mediated regulation of COX-2 expression in TCDD-exposed livers. Collectively, these observations suggest that cPLA_2_α has a distinct role in the development of fatty liver in TCDD-induced liver degeneration.

In agreement with our data (Table 3), hydroureter was previously observed in the urinary tracts of TCDD-exposed fetuses (Abbott et al. 1987; Bryant et al. 2001). These observations are in line with the current understanding of the etiology of TCDD-induced fetal hydronephrosis; ureteral lumens are anatomically obstructed by TCDD-induced hyperplasia, and the backpressure of urine expands the ureter and pyelocaliceal space of the kidney. This mechanism was independent of cPLA_2_α, as indicated by similar incidences of hydroureter and hydronephrosis in cPLA_2_α^+/+^ and cPLA_2_α^−/−^ fetuses (Tables 2, 3). However, cPLA_2_α was suggested to contribute to the progression of hydronephrosis because the incidence of the severest degree was lower in TCDD-exposed cPLA_2_α^−/−^ fetuses than that in cPLA_2_α^+/+^ fetuses. In contrast with TCDD-induced fetal hydronephrosis, TCDD-induced neonatal hydronephrosis is not accompanied by hydroureter or ureter obstruction (Nishimura et al. 2008; Yoshioka et al. 2016). The present data suggest that cPLA_2_α plays different roles in these fetal and neonatal types of hydronephrosis. Specifically, (1) cPLA_2_α is thought to be minimally involved in the onset of hydronephrosis in the fetal period (Table 2), but plays a predominant role in the neonatal period (Yoshioka et al. 2014). In addition, (2) mPGES-1 was not transcriptionally upregulated in TCDD-exposed fetal kidneys (Fig. 3d), but was prominently upregulated in TCDD-exposed neonatal kidneys in a cPLA_2_α-dependent manner (Yoshioka et al. 2014). Of note, mPGES-1 is indispensable for the onset of TCDD-induced hydronephrosis in neonatal mice (Yoshioka et al. 2012). Collectively, these data indicate that TCDD induces two distinct types of hydronephrosis in fetal and neonatal periods.

The canonical function of ligand-bound AHR involves the transcriptional activation of target genes through direct binding to their promoters, and the prototypical target is CYP1A1 (Mimura and Fujii-Kuriyama 2003). This function of AHR has been considered distinct from cytosolic enzymes, such as cPLA_2_α, because TCDD-induced upregulation of CYP1A1 expression was independent of cPLA_2_α (Dong and Matsumura 2008; Li et al. 2010; Sciullo et al. 2008; Yoshioka et al. 2014). On the other hand, some genes are reportedly upregulated by TCDD in a cPLA_2_α-dependent manner (Dong and Matsumura 2008, 2009; Yoshioka et al. 2014). TCDD-induced upregulation of IL-1β and TNF-α expression in the fetal kidneys was also dependent on cPLA_2_α (Fig. 3e, f). The underlying mechanisms of the cPLA_2_α dependency may involve the non-canonical AHR pathway, in which phosphorylated cPLA_2_α activates protein kinases upon Ca^2+^ influx independently of the transcriptional activity of AHR (Matsumura 2009). Besides these examples of potentially non-canonical gene expression upon TCDD exposure, the expression of CYP1A1 (Fig. 6a) and other AHR target genes (Supplementary Fig. 2) required cPLA_2_α for the full induction by TCDD in the adult liver, which was contrary to our expectations. Thus, the canonical functions of AHR could be modulated by various mechanisms, including those involving cPLA_2_α. Further studies of transactivation and other activities of AHR will reveal precise molecular mechanisms of responses to TCDD exposure.

Previous studies suggest the presence of unknown factor(s) that play pivotal role(s) in TCDD-induced fetal death. First, Holtzman rats are far more susceptible to TCDD than Sprague–Dawley rats, despite bearing identical primary structure of AHR (Kawakami et al. 2006). Second, although TCDD and 2-(19H-indole-39-carbonyl)-thiazole-4-carboxylic acid methyl ester (ITE) upregulate transcription of the prototypical AHR target CYP1A1 with similar potency, only the former induces fetal death in rats (Wu et al. 2014). These studies do not support the simple model in which the transactivation capacity of AHR determines the degree of fetal toxicity. Among candidate factors, cPLA_2_α is implicated because it plays essential roles in pregnancy and parturition (Bonventre et al. 1997; Leslie 2015), and because TCDD enhances the expression and activity of cPLA_2_α (Dong and Matsumura 2008; Kinehara et al. 2009). In the present study, the number of TCDD-exposed cPLA_2_α^−/−^ fetuses was significantly fewer than TCDD-exposed cPLA_2_α^+/+^ fetuses, suggesting that TCDD-exposed cPLA_2_α^−/−^ fetus numbers were decreased, or that TCDD-exposed cPLA_2_α^+/+^ fetus numbers were increased. The first possibility is plausible because the TCDD-exposed cPLA_2_α^−/−^ fetuses tended to be fewer than vehicle-control cPLA_2_α^−/−^ fetuses (*p* = 0.059), whereas TCDD-exposed cPLA_2_α^+/+^ fetuses were not more numerous than vehicle-control cPLA_2_α^+/+^ fetuses (*p* = 0.35). The second possibility is negated because additional fetuses cannot be expected after GD 12.5. Further investigations of the relationships between TCDD and cPLA_2_α will clarify the mechanisms of male specific reductions in live fetus numbers.

In conclusion, the present study revealed that cPLA_2_α participates in TCDD-induced body weight loss, lipid accumulation in the liver, fetal hydronephrosis, and changes in gene expression, and that the molecular basis of TCDD toxicities varies considerably between target tissues and life stages.

## Electronic supplementary material

Below is the link to the electronic supplementary material.

Supplementary material 1 (PDF 261 kb)
