# Supplementary material for: Roles of cytosolic phospholipase A2α in reproductive and systemic toxicities in 2,3,7,8-tetrachlorodibenzo-p-dioxin-exposed mice
Source: Arch Toxicol. 2017 Oct 17;92(2):789–801. doi: 10.1007/s00204-017-2081-z (PMC5818604; doi:10.1007/s00204-017-2081-z)
Supplement: Supplementary file 1 — Supplementary material 1 (PDF 261 kb) [file 204_2017_2081_MOESM1_ESM.pdf]

Supplementary Information

Roles of Cytosolic Phospholipase A2α in Reproductive and Systemic Toxicities in 2,3,7,8-Tetrachlorodibenzo-*p*-dioxin Exposed Mice

Nozomi Fujisawa, Wataru Yoshioka[*2], Hiroyuki Yanagisawa, and Chiharu Tohyama[*1]

*To whom correspondence should be addressed.
1. E-mail: tohyamac-tky@umin.org
2. E-mail: yoshioka-w@umin.ac.jp

Supplementary Table 1. Sequences of primers used for qPCR

Target Gene		Primer Sequences (5′ to 3′)
AHRR	Forward	CAG GGC AGA CAT TGT GGT TA
	Reverse	CTC CAT TGC TCT TTC CTG CT
COX-2	Forward	TGT GAA CAA TCA AAC AAA ATG ATG
	Reverse	GCG TAA ATT CCA ACA GCC TAA GT
cPLA$_2$*α*	Forward	AGC ATT CAA AAG GCT TCA CG
	Reverse	GGG AAA CAG AGC AAC GAG AT
Cyclophilin B	Forward	GAC TTC ACC AGG GGA GAT GG
	Reverse	TGT GAG CCA TTG GTG TCT TTG
CYP1A1	Forward	GGC ACC TCT GTT CAC CCT A
	Reverse	GAA TCT CTC CCT CTG TTC TTG
CYP1B1	Forward	TGC CTG CCA CTA TTA CGG AC
	Reverse	GGT CCC TCC CCA CAA CCT
F4/80	Forward	TGC ATC TAG CAA TGG ACA GC
	Reverse	GCC TTC TGG ATC CAT TTG AA
IL-1β	Forward	CAA GGA GAA CCA AGC AAC GA
	Reverse	GCC GTC TTT CAT TAC ACA GGA
mPGES-1	Forward	CTC AAG CCC TGC TAC CAC A
	Reverse	GGC CTC AGA CAA GAG ACC AT
miR-101a	Forward	GCC TAC AGT AGT GTG ATA AC
Nqo1	Forward	AGA CCT TGC TTT CTA TCA CCA CT
	Reverse	AGA CCT GGA AGC CAC AGA AAC
TNF-α	Forward	CAC CAC CAT CAA GCA CTC AA
	Reverse	GAC AGA GGC AAC CTG ACC AC
U6 snRNA	Forward	CGC TTC GGC AGC ACA TAT ACT AA

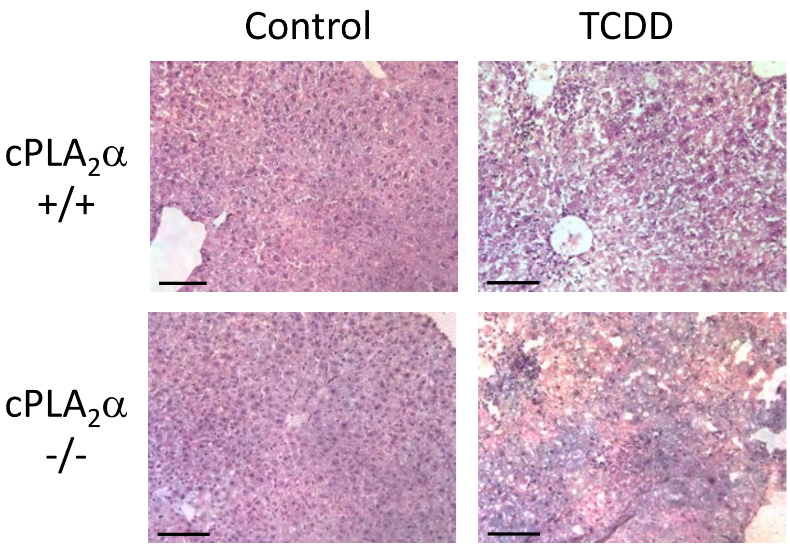

Supplementary Figure 1. Representative photographs of hematoxylin and eosin staining of livers of adult male mice; Bar = 100 μm. cPLA$_2$α$^{+/+}$ and $^{-/-}$ mice were intraperitoneally injected TCDD at a dose of 0 (Control) or 50 μg/kg body weight on Day 0, and the livers were analyzed on Day 10.

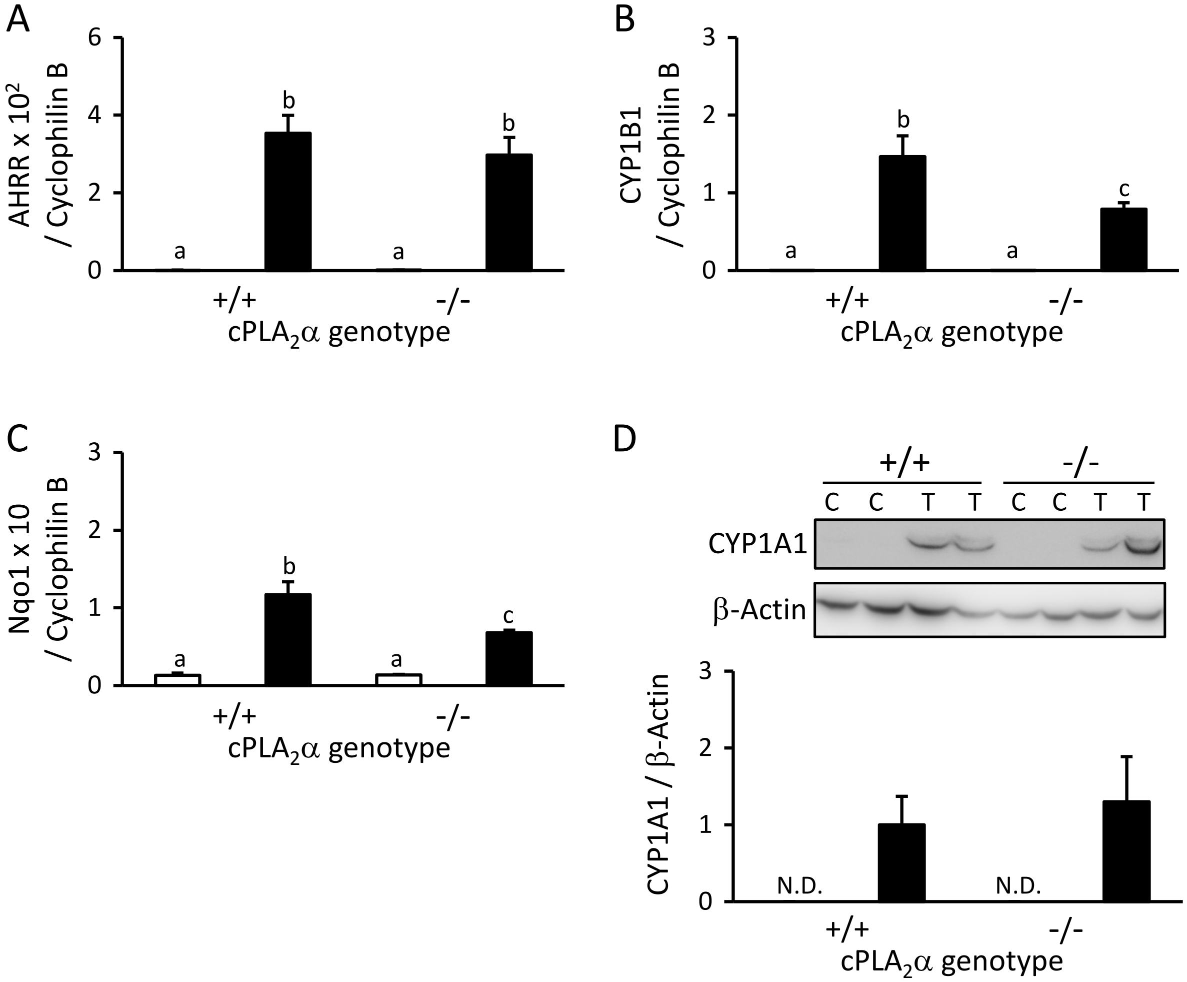

Supplementary Figure 2. AHRR (A), CYP1B1 (B), and Nqo1 (C) mRNA expression, and CYP1A1 protein expression (D) in livers of cPLA$_2\alpha^{+/+}$ and cPLA$_2\alpha^{-/-}$ adult male mice at 8 days post administration of TCDD (50 μg/kg body weight) or vehicle. Black and open histogram indicate TCDD and vehicle treatment, respectively. Bars indicate means ± SEM (n = 4). N.D.: not detected. Histograms with different letters indicate significant differences by Tukey post hoc test (A-C) and Welch's *t*-test (D).
